# Primary Retroperitoneal Mucinous Carcinoma with Carcinosarcomatous Mural Nodules: A Case Report with Emphasis on Its Histological Features and Immunophenotype

**DOI:** 10.3390/diagnostics10080580

**Published:** 2020-08-11

**Authors:** Sujin Park, Hyun-Soo Kim

**Affiliations:** Department of Pathology and Translational Genomics, Samsung Medical Center, Sungkyunkwan University School of Medicine, Seoul 06351, Korea; sujin423.park@samsung.com

**Keywords:** retroperitoneum, mucinous carcinoma, carcinosarcoma, mural nodule

## Abstract

Mucinous carcinoma of the retroperitoneal origin is extremely rare. The existence of mural nodules in association with retroperitoneal mucinous carcinoma is an even rarer condition and indicates a worse prognosis. We present a case of primary retroperitoneal mucinous carcinoma with carcinosarcomatous mural nodules in a 27-year-old woman. We found a histological spectrum of mucinous tumors encompassing a mucinous borderline tumor, microinvasive carcinoma, and overt carcinoma with an expansile invasive pattern. The mural nodules had two morphological components. The sarcomatous component consisted of diffusely proliferating pleomorphic spindle or polygonal cells, while the carcinomatous component exhibited infiltrative glands showing a complex and cribriform architecture as well as distorted and poorly formed small glands. The carcinomatous component, comprising approximately 20% of the entire tumor volume of the mural nodules, was randomly distributed within the sarcomatous component. In a few areas, the carcinomatous component transformed and merged into the sarcomatous component. Immunostaining revealed a mutually exclusive pattern of expression of cytokeratin and vimentin in the carcinomatous and sarcomatous components, respectively, supporting the presence of a dual tumor cell population and confirming the diagnosis of carcinosarcoma. In summary, our case exhibited a histological spectrum of mucinous tumors and a metaplastic transformation from the carcinomatous to sarcomatous component in mural nodules. The immunostaining results of a mutually exclusive expression pattern of epithelial and mesenchymal markers confirmed the histological evidence of a dual population. Although rare, the specific histological features and immunophenotype are helpful in establishing the diagnosis of carcinosarcomatous mural nodules. Since the pathogenetic mechanism and treatment strategies for primary retroperitoneal mucinous carcinoma remain unclear, pathologists have an essential role to play in correctly evaluating the presence of mural nodules and determining their nature, to be later utilized to predict patients’ outcomes and provide appropriate treatment.

## 1. Introduction

Mucinous carcinoma of the retroperitoneum is a very uncommon condition [1]. Mural nodules associated with primary retroperitoneal mucinous carcinoma are even rarer. The mural nodules are classified histologically as sarcoma-like mural nodules, anaplastic carcinoma, true sarcoma, and carcinosarcoma [2]. A sarcoma-like mural nodule is a misleading benign entity, which may be associated with benign, borderline, or malignant mucinous neoplasms of the ovary and retroperitoneum [2,3]. It should be distinguished from other malignant mural nodules for proper management [3,4]. We herein present a very rare case of primary retroperitoneal mucinous carcinoma with carcinosarcomatous mural nodules occurring in a 27-year-old woman. We provide a detailed description of its histological features and immunophenotype. A comprehensive clinicopathological analysis of primary retroperitoneal mucinous carcinoma with carcinosarcomatous mural nodules will serve to improve the current understanding of this rare entity and help pathologists to make a correct diagnosis.

## 2. Case Report

### 2.1. Clinical Presentation

**Figure 1:**
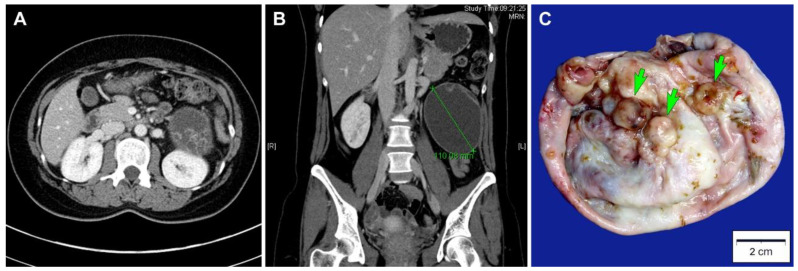
Imaging and gross findings. (**A**) Abdominopelvic computed tomography scan in axial view, which revealed a thick-walled cystic mass in the left retroperitoneal space. The mass had some daughter cysts. (**B**) Abdominopelvic computed tomography scan in coronal view, which revealed a well-circumscribed, ovoid unilocular cystic mass with a diameter of 11 cm. (**C**) Grossly, the inner surface of the mass showed some round-to-ovoid, variegated mural nodules (green arrows), measuring up to 1.2 cm in the greatest dimension.

A 27-year-old Korean woman presented with abdominal pain. She had no previous gynecological history. An abdominopelvic computed tomography scan revealed a well-circumscribed, thick-walled cystic mass located between the left kidney and descending colon with a diameter of 11 cm (Figure 1A). The unilocular cystic mass in the left retroperitoneal space had a number of daughter cysts (Figure 1B). No abdominopelvic seeding or lymph node enlargement was identified. Based on the preoperative diagnosis of a primary retroperitoneal tumor, surgical mass excision was performed. The bilateral adnexa, kidneys, liver, and pancreas were unremarkable. A simple tumor excision was performed without intraoperative rupture.

The patient did not receive any further treatment such as postoperative chemotherapy or radiation therapy. Four months after surgery, she is well, without evidence of recurrent disease or distant metastasis.

### 2.2. Pathological Findings

The resected specimens were fixed in 10% neutral-buffered formalin and embedded in paraffin blocks. From each formalin-fixed, paraffin-embedded block, 4 μm sections were cut and stained with hematoxylin and eosin. All hematoxylin- and eosin-stained slides were examined microscopically. The most representative hematoxylin- and eosin-stained slides were chosen for immunostaining. A board-certified pathologist made a final pathological diagnosis.

**Figure 2:**
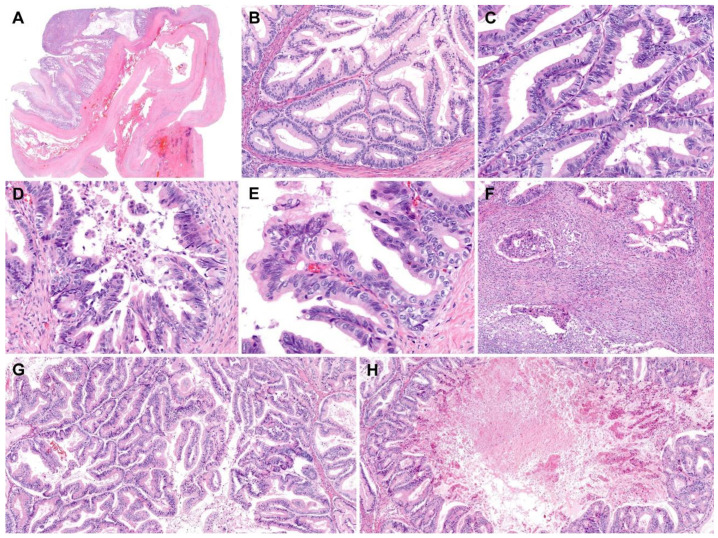
Histological features of retroperitoneal mucinous carcinoma. (**A**) The thick fibrous wall showed mural hemorrhage. (**B**) Areas showing glandular proliferation were characterized by nuclear stratification and low-to-intermediate-grade nuclear atypia without stromal invasion, compatible with a mucinous borderline tumor. (**C**–**E**) In several foci, (**C**) high-grade nuclear atypia, (**D**) a micropapillary pattern, a loss of polarity, and (**E**) intraluminal papillary projections were noted. (**F**) Irregularly shaped cellular clusters and cribriform glands infiltrated the stroma, indicating microinvasion. There were associated stromal inflammatory infiltrates and desmoplastic reactions. (**G**,**H**) In addition to mucinous borderline tumors and microinvasive mucinous carcinomas, areas characterized by confluent glandular proliferation without intervening stroma were present, compatible with mucinous carcinomas with an expansile invasive pattern. A large, proliferating gland exhibited a cribriform architecture. Its lumen was extensively dilated and contained necrotic debris. Staining method: (**A**–**H**), hematoxylin and eosin. Original magnification: (**A**), 5×; (**B**), 40×; (**C**), 100×; (**D**), 100×; (**E**), 150×; (**F**), 40×; (**G**), 40×; (**H**), 20×.

Grossly, the inner surface of the unilocular cystic mass showed some nodular elevations measuring up to 1.2 cm (Figure 1C). Histologically, the tumor consisted predominantly of a thick fibrous cystic wall with multiple areas of mural hemorrhage and chronic inflammation (Figure 2A). A confluent proliferation of pseudostratified mucin-containing columnar epithelium was noted (Figure 2B). In some areas, extensively dilated glandular lumina contained an admixture of mucins, blood, and numerous inflammatory cells. The neoplastic glands varied in size and shape. The lining epithelium was partially denuded, and the subepithelial stroma was characterized by severe inflammation, foreign body reaction, and fibrosis due to mucin spillage. The mucin-containing epithelium demonstrated a spectrum of borderline and malignant morphologies. Most parts of the tumor showed histological features identical to those of ovarian mucinous borderline tumors; however, several areas showed high-grade nuclear atypia including severe enlargement, pleomorphism, prominent nucleoli, increased mitotic activity, and frequent atypical mitotic figures (Figure 2C). In addition to high-grade nuclear atypia, a loss of epithelial polarity, intraluminal papillary epithelial projections, a micropapillary growth pattern, and a cribriform architecture—features characteristic of intraepithelial carcinoma—were also present (Figure 2D,E). A few microinvasive foci, showing tumor cells that formed clusters or papillae or that were scattered individually in inflammatory stroma, were also noted (Figure 2F). We also identified some areas showing mucinous carcinomas with an expansile invasive pattern (Figure 2G), with little or no intervening stroma and extensively dilated glandular lumina containing necrotic debris (Figure 2H). Taken together, this retroperitoneal tumor was diagnosed as a mucinous carcinoma showing a histological spectrum of a mucinous tumor, including a mucinous borderline tumor associated with multifocal microscopic intraepithelial carcinoma, microinvasive mucinous carcinoma, and overt mucinous carcinoma with an expansile invasive pattern.

**Figure 3:**
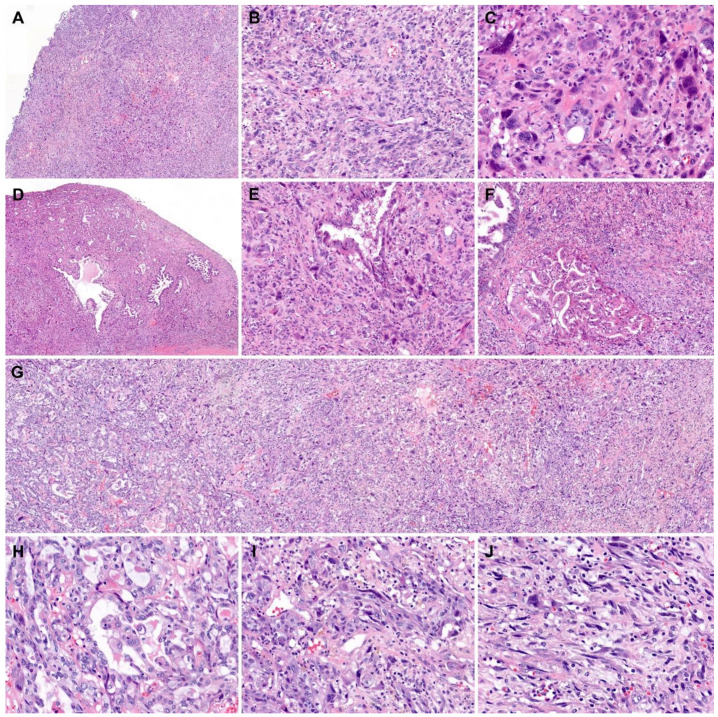
Histological features of mural nodules associated with retroperitoneal mucinous carcinoma. (**A**) The mural nodules showed a diffuse proliferation of pleomorphic tumor cells, forming large, solid cellular sheets. (**B**) The spindle-shaped or polygonal tumor cells were arranged haphazardly. (**C**) Bizarre or multinucleated tumor cells were noted. (**D**) In some areas, variably sized, irregularly shaped glands were randomly distributed within the sarcomatous component. (**E**) An angulated tumor gland was embedded within the sarcomatous component. (**F**) Two large glands, which showed a complex, cribriform architecture, were present. (**G**–**J**) Sarcomatous transformation. The carcinomatous component displayed (**H**) a moderately differentiated adenocarcinoma (left one-third of image **G**) transformed through (**I**) a poorly differentiated carcinoma (middle one-third of image **G**) into (**J**) a sarcoma (right one-third of image **G**). Staining method: (**A**–**J**), hematoxylin and eosin. Original magnification: (**A**), 10×; (**B**), 100×; (**C**), 400×; (**D**), 10×; (**E**), 100×; (**F**,**G**) 40×; (**H**–**J**), 200×.

We also thoroughly examined the histological features of the mural nodules. All the tumor tissues obtained from the mural nodules were submitted for histological examination (Figure 3A). The mural nodules consisted mainly of diffusely proliferating spindle-shaped or polygonal pleomorphic cells (Figure 3B). Bizarre, multinucleated tumor cells were noted occasionally (Figure 3C). Up to 19 mitotic figures were counted per 10 high-power fields, and atypical mitoses were often identified. Some areas showed neoplastic glandular proliferation characterized by a complex and cribriform architecture (Figure 3D), as well as poorly formed small and irregularly shaped glands. This carcinomatous component was distributed randomly within the sarcomatous component consisting of spindle-shaped or polygonal pleomorphic cells (Figure 3E,F). In a few areas showing both components, the carcinomatous component appeared to transform and merge into the sarcomatous component (Figure 3G–J), which was diagnostic of carcinosarcoma. The carcinomatous component comprised approximately 20% of the entire tumor volume of the mural nodules. No heterologous component was identified.

### 2.3. Immunostaining Results

Immunohistochemical staining was performed using a compact polymer method (Bond Polymer Refine Detection kit; Leica Biosystems, Newcastle, UK). Four-micrometer formalin-fixed, paraffin-embedded sections were incubated with primary antibodies against pan-cytokeratin, cytokeratin 7, epithelial membrane antigen, cytokeratin 20, vimentin, caudal-type homeobox transcription factor 2, and Ki-67.

**Figure 4:**
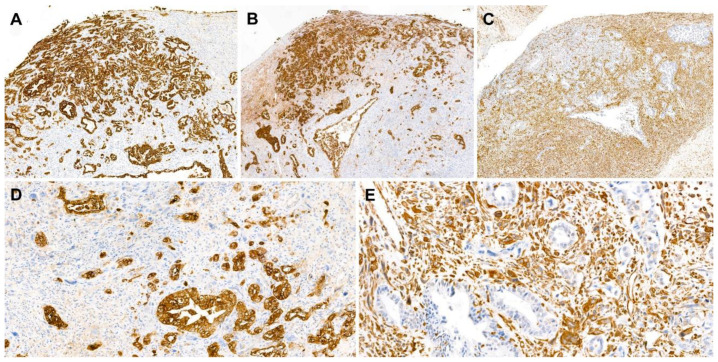
Immunostaining results. (**A**,**B**) The carcinomatous component was highlighted using (**A**) cytokeratin 7 and (**B**) epithelial membrane antigens. (**C**) The sarcomatous component reacted with vimentin. (**D**,**E**) In high-power view, the carcinomatous and sarcomatous components showed mutually exclusive immunoreactivity to (**D**) pan-cytokeratin and (**E**) vimentin, respectively. Staining method: (**A**–**E**), polymer method. Original magnification: (**A**–**C**), 40×; (**D**,**E**), 200×.

The carcinomatous component tested diffusely and strongly positive for pan-cytokeratin, cytokeratin 7 (Figure 4A), and epithelial membrane antigen (Figure 4B). The expression of cytokeratin 20 in the carcinomatous component was focal but strong. The sarcomatous component tested diffusely and strongly positive for vimentin (Figure 4C) but negative for cytokeratin 7, epithelial membrane antigen, and cytokeratin 20. Both components tested negative for caudal-type homeobox transcription factor 2. The Ki-67 labeling index was approximately 40% in both components. The mutually exclusive expressions of pan-cytokeratin (Figure 4D) and vimentin (Figure 4E) in the carcinomatous and sarcomatous components, respectively, were diagnostic of carcinosarcoma.

## 3. Discussion

Based on the presence of a mucinous carcinoma that morphologically corresponded to that of ovarian origin, mural nodules exhibiting histological features typical of carcinosarcoma, and a mutually exclusive expression pattern of cytokeratin and vimentin, we made a final pathological diagnosis of primary retroperitoneal mucinous carcinoma with carcinosarcomatous mural nodules. Primary retroperitoneal mucinous tumors are a very rare condition. Similar to mucinous ovarian tumors, mucinous tumors of the retroperitoneum are classified histologically into mucinous cystadenomas, mucinous borderline tumors, and mucinous carcinomas [5,6]. To the best of our knowledge, 62 cases of retroperitoneal mucinous carcinoma have been reported in the English literature to date [1]. Most of the patients were women, and the greatest diameter of the tumors ranged from 7–31 cm. The tumors presented as unilocular or multilocular cystic masses filled with mucous material [5]. Since many different types of neoplastic lesions present as retroperitoneal cystic masses [7], it may be impossible to obtain definitive diagnoses of these lesions from preoperative imaging studies only. Although rare, primary retroperitoneal mucinous carcinomas should be included in the differential diagnosis of retroperitoneal cystic masses, and a thorough pathological examination is essential for establishing an accurate diagnosis and appropriate management.

Fifteen cases of retroperitoneal mucinous borderline tumors or carcinomas associated with mural nodules have been reported to date [8]. The mural nodules are histologically classified into benign reactive and malignant lesions. The former are known as sarcoma-like mural nodules, and malignant lesions are categorized further into anaplastic carcinomas, true sarcomas, and carcinosarcomas. Sarcoma-like mural nodules derive from undifferentiated mesenchymal cells located beneath mucinous epithelium. These undifferentiated mesenchymal cells proliferate in response to neoplastic processes in the overlying mucinous epithelium, hemorrhage, or mucin spillage [9,10]. Sarcoma-like mural nodules are observed adjacent to areas of intraepithelial neoplastic transformation, morphologically characterized by cellular crowding and nuclear stratification and enlargement, explaining their frequent association with mucinous borderline tumors or carcinomas [5]. Sarcoma-like mural nodules are typically histologically characterized by good circumscription, a heterogeneous cell population, epulis-type multinucleated giant cells, degenerative changes such as karyorrhexis and bizarre nuclei, a hemorrhagic or inflammatory background, and a lack of carcinomatous or sarcomatous neoplastic components. By contrast, true sarcomas are typically poorly circumscribed tumors consisting of spindle-shaped or polygonal cells with pleomorphic, hyperchromatic nuclei and conspicuous nucleoli. Multiple areas of coagulative tumor cell necrosis are also present. The diagnosis of carcinosarcomatous mural nodules is based on histological evidence of dual carcinomatous and sarcomatous components.

Surgical mass excision is the most widely accepted treatment option for primary retroperitoneal mucinous carcinoma [11], but some authors have stated that a total hysterectomy (TH) with bilateral salpingo-oophorectomy (BSO) should be performed for patients who do not want to preserve fertility [12]. Liu et al. [8] reviewed the previously published 66 cases of primary retroperitoneal mucinous carcinoma. Fourteen patients were diagnosed as having mucinous borderline tumors, and all of these patients received a simple tumor excision with or without postoperative chemotherapy. None of them was treated with a TH with BSO or died of this disease. Fifty-two patients had mucinous carcinoma components, and 13 of them received a TH with BSO. Of these, one died of disease during follow up.

It has been reported that patients whose tumors were suspected to be spilled or ruptured during surgery received chemotherapy following surgical excision [13]. Additionally, in cases with metastasis or local recurrence, postoperative chemotherapy was performed. Liu et al. [8] reported that one patient with a mucinous borderline tumor received chemotherapy after surgery but developed disease recurrence. Eleven of the 52 patients with mucinous carcinoma components received postoperative chemotherapy, three developed recurrence, and three died. Since there is no established evidence of the efficacy of postoperative chemotherapy, further investigation is necessary to determine whether the adjuvant therapy is effective for patients with primary retroperitoneal mucinous carcinomas.

Previous studies showed that the presence of mural nodules in association with a retroperitoneal mucinous carcinoma indicates a worse prognosis [14,15]. Another study also reported that among 12 patients with mural nodules, three died of disease and one developed recurrence. The rate of death in patients with retroperitoneal mucinous carcinomas associated with mural nodules (25.0%) was higher than in those without mural nodules (12.5%) [8]. These data suggest that primary retroperitoneal mucinous carcinomas with mural nodules may have aggressive prognoses.

In summary, we have presented a rare case of primary retroperitoneal mucinous carcinoma with carcinosarcomatous mural nodules. Our case exhibited a histological spectrum of mucinous tumors including a mucinous borderline tumor, microinvasive mucinous carcinoma, and mucinous carcinoma with an expansile invasive pattern and a metaplastic transformation from the carcinomatous to sarcomatous component in mural nodules, confirmed by a mutually exclusive expression pattern of epithelial and mesenchymal markers. The specific histological features and immunophenotype of the mural nodules are helpful in establishing the diagnosis. An accurate diagnosis is critical in view of its significant impact on the management of these rare tumors.
